# Improving berry quality and antioxidant ability in ‘Ruidu Hongyu’ grapevine through preharvest exogenous 2,4-epibrassinolide, jasmonic acid and their signaling inhibitors by regulating endogenous phytohormones

**DOI:** 10.3389/fpls.2022.1035022

**Published:** 2022-12-02

**Authors:** Jiajia Li, Hafiz Umer Javed, Zishu Wu, Lei Wang, Jiayu Han, Ying Zhang, Chao Ma, Songtao Jiu, Caixi Zhang, Shiping Wang

**Affiliations:** ^1^ Department of Plant Science, School of Agriculture and Biology, Shanghai Jiao Tong University, Shanghai, China; ^2^ College of Chemistry and Chemical Engineering, Zhongkai University of Agricultural Engineering, Guangzhou, China; ^3^ Grape and Wine Institute, Guangxi Academy of Agricultural Sciences, Nanning, Guangxi, China

**Keywords:** exogenous phytohormones, berry quality, antioxidant ability, endogenous phytohormones, correlation analysis, grapevine

## Abstract

Grape berries contain a variety of metabolites, such as anthocyanins, sugars, fatty acids, and antioxidants. Endogenous phytohormones strongly influence these metabolites, which regulate berry quality improvement. In this study, we evaluated the effects of 2,4-epibrassinolide (EBR, brassinolide (BR)-like growth regulator), jasmonic acid (JA), and their signaling inhibitors brassinazole (Brz), and sodium diethyldithiocarbamate (DIECA) on berry quality and antioxidant ability. Overall, the pre-harvest application of 0.5 mg L^-1^ EBR and 100 μmol L^-1^ JA significantly influences the quality of the grape berry. Results showed that EBR was superior to other treatments at enhancing the content of different metabolites, including anthocyanins, fructose, glucose, and a variety of fatty acids, in grapes. EBR and JA also enhanced the synthesis of gibberellin_3_ (GA_3_), cytokinin (CTK), salicylic acid (SA), JA, methyl jasmonate (MeJA), BR, and abscisic acid (ABA), while inhibiting the synthesis of auxin (IAA). Most genes related to BR/JA and anthocyanins/sugars/fatty acids biosynthesis were up-regulated. The effects of Brz and DIECA on the grape berry quality were totally reversed throughout the study, as shown by EBR and JA. According to correlation analysis, EBR and JA have a beneficial positive interaction that promotes the formation of strong coherences in grape berries between ABA/IAA/ZT-fruit expansion, BR/JA/MeJA/GA_3_/ZR-biochemical characteristics development, JA/MeJA/ABA/GA_3_/SA/ZR-antioxidant capacity enhancement, and JA/MeJA/IAA/GA_3_/ZT/ZR-fatty acids accumulation. In this regard, we concluded that preharvest exogenous 0.5 mg L^-1^ EBR and 100 μmol L^-1^ JA is a successful way to improve grape berry quality.

## Introduction

Grapevine (*Vitis vinifera* L.), originating in western Asia and cultivated worldwide, is a crucial horticultural crop (Ramos-Madrigal et al., 2019). Due to their great taste and high nutritional content, grapes are popular with people and are widely planted worldwide (Bouby et al., 2021). The nutrient value of grape berries could be characterized by various parameters, such as berry size, texture, pericarp color, solid acid ratio, and aroma composition. Additionally, the content of anthocyanins, sugar, organic acids, fatty acids, and terpenes directly reflects the nutritional quality of grape berries and extensively contributes to fruit coloring and flavor formation (Pott et al., 2020). Furthermore, the fruit metabolites involved in stress resistance, such as flavonoids, polyphenols, and ascorbic acid, change significantly as the fruit develops and ripe (Pastore et al., 2013; Martín-Tornero et al., 2020; Dalicuba et al., 2021). Molecular plant breeding takes a long time to improve grape berry quality. Thus, the researcher has focused on using non-toxic regulators (exogenous phytohormones; plant growth regulators) to spray horticultural crops in order to enhance fruit appearance and quality quickly (Sun et al., 2020a; Li et al., 2021a).

BR, a sterol phytohormone, was recognized as the sixth-largest phytohormone due to its beneficial effects on plant growth and stress resistance, even at a very low concentration (Sun et al., 2020b; Nakagawa et al., 2021). Extensive studies have focused on how BR affects horticultural crop development and fruit quality improvement. BR is responsible for the enhancement of sugar accumulation in sweet cherries (Roghabadi and Pakkish, 2014), as well as the significantly increased total anthocyanins (33.7%), total phenols (25.4%), and total flavonoids (23.9%) in pomegranate fruits (Tadayon and Hosseini, 2021). Exogenous BR (100 μmol L^-1^) treatment also changed the aroma components in five-year-old ‘Kyoho’ grape berries and increased the contents of terpenes, particularly β-pinene and D-limonene. Furthermore, it has an effect on the 3-hydroxy-3-methyl glutaryl coenzyme A reductase (HMGR) activity, which is recognized as a key enzyme for the biosynthesis of terpenes and is primarily found in the berry skin. Its activity continued at high levels during the post-harvest storage period to avoid fruit discoloration (Zheng et al., 2020). In-depth molecular research confirmed that transcription factors from the MYB, WRKY, and SUC families bind closely to Brassinosteroid resistant 1/2 (BZR1/2), Brassinosteroid Insensitive 1(BRI1) associated kinase receptor 1 (BAK1), which is recognized as a receptor protein responsible for BR signaling. BR also contributed to the biosynthesis of anthocyanins in tomatoes, terpenes volatilization in grapes, and the accumulation of sugars in pears (Shen et al., 2017; Sun et al., 2019; Yang et al., 2021a).

JA was reported to play an essential role in inducing stomatal closure, inhibiting rubisco biosynthesis, affecting the absorption of N and P, and influencing the transport of glucose as well as other organic substances (El-kenawy, 2018; Gomi, 2020). It is noteworthy that the effect of JA on horticultural crop quality has been extensively addressed. It considerably promotes apple color and the accumulation of sugars in peaches, which finally leads to a significant improvement in the fruit’s commercial and nutritional values (Gao et al., 2021; Zhao et al., 2021). Wang et al. (2022) reported that interaction between MdERF1B and MdJAZ5/10 was involved in activating the expression of MdMYC2, thus inducing the considerable accumulation of anthocyanin in apple fruits. Furthermore, the exogenous application of JA promoted ethylene (ETH) production by increasing the abundance of ACS1 mRNA, along with a remarkable increase in soluble sugar content and protecting from the chilling injury of peaches (Zhao et al., 2021). Chen et al. (2019) reported that MeJA could regulate fatty acids in phospholipids by activating the JA-mediated C-repeat-binding factor pathway, which quickly and efficiently helped reduce the degree of chilling injury in peach fruit. A molecular study revealed that pathogens and 100 mmol L^-1^ MeJA could affect *VvDOF3* expression. Moreover, the overexpressed *VvDOF3* showed lower powdery mildew symptoms than WT plants and increased *PDF1.2* (JA biosynthesis-related gene) expression (Yu et al., 2019). In previous studies, JA and BR both were found to be effective phytohormones that play significant roles in plant growth and fruit quality on different horticultural crops. However, only a little research work has been done specifically on grapes, and more extensive research is required to fully understand the mechanism.

Previous studies revealed that 0.4 and 0.6 mg L^-1^ EBR significantly increased proanthocyanidins accumulations and accelerated soluble sugars metabolism in ‘Cabernet Sauvignon’ grape and ‘Merlot’ grape, respectively (Xu et al., 2015; Yan et al., 2022). On the other hand, 100 μmol L^-1^ JA significantly affected the berry enlargement of ‘Blueain’ blueberries (Liu et al., 2022). Thus, it was found that 0.4 to 0.6 mg L^-1^ EBR and 100 μmol L^-1^ JA were effective for enhancing grape berry quality in terms of its physical appearance, physicochemical qualities, and sensory attributes. Therefore, a comprehensive study against the recently developed ‘Ruidu Hongyu’ grapevine was designed using 0.5 mg L^-1^ and 100 μmol L^-1^ concentrations of EBR and JA, respectively. After that, we assess how EBR and JA, along with their signaling inhibitors (Brz and DIECA), affect the quality of grape berries, as well as metabolites (anthocyanins, fructose, glucose, various fatty acids) and antioxidant enzymatic activity (guaiacol peroxidase (POD), superoxide dismutase (SOD), catalase (CAT), and polyphenol oxidase (PPO)). Further, a molecular study at the transcriptional level is carried out to understand the mechanism, and a correlation analysis is performed to predict the potential endogenous phytohormones crosstalk.

## Materials and methods

### Experimental design and material cultivating

A six-year-old ‘Ruidu Hongyu’ grapevine, a new grape variety with pink fruit and early-ripening characteristics, a red bud mutant of ‘Ruidu Xiangyu’ (‘Jingxiu’ *×* ‘Xiangfei’), was selected as the test material. It was planted in the arch shed at Grape and Wine Institute, Guangxi Academy of Agricultural Sciences (107°45’ E, 22°13’ N, NanNing, Guangxi, China). There were six different treatments group created, including the control, 0.5 mg L^-1^ EBR, 0.5 mg L^-1^ EBR + 1 mg L^-1^ Brz (BR signaling inhibitor), 100 μmol L^-1^ JA, 100 μmol L^-1^ JA + 10 mmol L^-1^ DIECA (JA signaling inhibitor), and 0.5 mg L^-1^ EBR + 1 mg L^-1^ Brz + 100 μmol L^-1^ JA + 10 mmol L^-1^ DIECA. The same water and fertilizer conditions were applied to all treatment groups. Seven days before the veraison stage (DAA 30, where DAA indicated days after anthesis), three grapevines were selected in each treatment based on vine growth conditions. The application of exogenous growth regulators (EBR, Brz, JA, and DIECA) was then applied to eight healthy clusters of grapes from each grapevine that contained whole-grain grapes. Six sampling stages were established: 0 days after treatment (DAA 30), 10 days after treatment (DAA 40), 20 days after treatment (DAA 50), 30 days after treatment (DAA 60), 40 days after treatment (DAA 70), and 50 days after treatment (DAA 80). The experimental graphic abstract was designed and shown in **Figure S1**.

At each sampling point, 300 fruit berries were selected from the respective treated group that had a complete morphology and were free of diseases. These berries were then quickly transported with enough dry ice to the lab at the Department of Plant Science, School of Agriculture and Biology, Shanghai Jiao Tong University (121°29’ W, 31°11’ N, Shanghai, China), where further analysis would be conducted. Firstly, fruit size and single-berry weight were measured from respective applications. Then grape berries were thoroughly ground with liquid nitrogen by a vacuum grinder (IKA, GER) and kept at -80°C for the subsequent quantification of fruit quality-related parameters (reducing sugar, soluble sugar, total polyphenolic, flavonoid, soluble protein, ascorbic acid, anthocyanins), as well as stress resistance parameters (malondialdehyde (MDA), proline (Pro), POD, PPO, CAT, SOD).

### Physiological parameters measurements

#### Berry weight, longitudinal and transverse diameters

The fruit appearance parameters of five grape berries were measured, and the weight of a single berry was determined using an analytical balance (Sartorius, German). Longitudinal and transverse diameters were measured using a vernier caliper (Mitutoyo, TKY, Japan). All experiments were subjected to three biological repetitions.

### Biochemical properties measurements

#### TSS and TA

Total soluble solid (TSS) was measured by a refractometer (OWELL, Hangchow, CHN) using 1 mL of juice from extruded grape berries. Titratable acid (TA) was quantified from 5 mL of grape juice using a potentiometric titrator (HAINENG, SZ, CHN). All experiments were subjected to three biological repetitions. Images of grape berries phenotypes and experimental equipment were all obtained using a Canon camera (EOS 80D, Otamori Prefecture, Japan).

#### Total Polyphenolic content, total flavonoid content and total anthocyanins content

The TPC, TFC, and TAC were measured using Folin–Ciocalteu’s reagent extraction method, vanillin colorimetric assay, and the pH-differential method. In the beginning, 1 g berry sample was ultrasonically extracted at 45°C for 40 minutes with 80% methanol and 1% hydrochloric acid (solid-liquid ratio: 1:30). Then, the supernatant was collected for further detections of TPC, TFC, and TAC. In order to measure TPC, the supernatant was mixed with 3 mL of Folin-reagent Ciocalteu’s (0.2 M) and 2.4 mL of sodium carbonate (0.7 M) solution, which was then left for two hours in the dark. At 760 nm, the absorbance value was recorded, and TPC was then calculated using gallic acid equivalents (GAE). Regarding TFC, 0.2 mL of the supernatant solution was reacted for 6 minutes with 60 μl of 5% sodium nitrite solution. Following that, 1 M sodium hydroxide (0.8 mL) and ethanol (3.88 mL) were added progressively, and the mixture was then left for 15 minutes. The TFC absorbance was noted at 510 nm, and concentration was calculated using (+)-catechin hydrate as a standard. In the case of TAC, 2.5 mL of supernatant was diluted to 10 mL using 0.4 M sodium acetate buffer (pH = 4.5) and 0.025 M potassium chloride buffer (pH = 1), respectively. The absorbance of the two solutions was then measured at both wavelengths (520 and 700 nm), and the TAC content was converted to cyanidin-3-glucoside (C3G) equivalents.

#### Soluble protein and ascorbic acid contents

Soluble protein was quantified using the Coomassie Bright Blue assay in accordance with Singleton and Rossi’s (1965) methodology. The soluble protein concentration was calculated to be 595 nm and was represented in terms of g kg^-1^. According to the detailed method of Bonilla et al. (1999), the content of ascorbic acid in grape berries was determined and expressed as g kg^-1^ FW after absorbance was measured at 534 nm wavelength.

### Antioxidant enzymes activity detections

The stress resistance parameters such as MDA, Pro, POD, SOD, CAT, and PPO were assessed using a spectrophotometer (Macy, SHH, CHN). These measurements were done by slightly modifying Nahakpam and Shah’s (2011) procedure.

#### MDA and pro contents

MDA extraction method was as follows: 1 g of freeze-dried grape berry powder was added to a 10 ml tube containing 5 mL of 100 g L^-1^ TCA, and the mixture was centrifuged at 4°C for 20 minutes at 10,000 g. Then, take 2 mL of supernatant and mix in 2 mL of a 0.67% TBA solution. The reaction mixture was centrifuged once more (4°C, 10,000 × g) after being heated in a boiling water bath for 20 minutes. MDA value was determined at 450, 532, and 600 nm and expressed as µmol kg^-1^.

The following was the pro extraction method: Measuring 2 g of freeze-dried grape berry powder, adding 5 mL of the sulfosalicylic acid solution, and heating the mixture in a boiling water bath for 10 minutes. After being cooled to room temperature (25°C), the sample was centrifuged at 10,000 × g for 15 min. Subsequently, 2 mL of supernatant, 2 mL of glacial acetic acid, and 3 mL of acidic ninhydrin reagent were adequately mixed. Then, after cooling, 4 mL of toluene was added after the reaction had taken place in a boiling water bath for 30 minutes. The mixture was shaken for an additional 30 seconds, after which it was allowed to stand for 10 minutes. At 520 nm, the absorbance of the supernatant was measured, and the concentration was expressed as mg kg^-1^.

#### CAT and SOD enzymes activities

CAT and SOD extraction was carried out by mixing 5 g of freeze-dried grape berry powder with 5 mL of extraction solution (5 mmol L^-1^ DTT, 5% PVP, 0.1 mol L^-1^ sodium phosphate buffer, pH = 7.5). The supernatant was obtained as an enzyme extraction solution by centrifuging the sample at 12,000 × g for 30 minutes at 4°C. The CAT’s enzymatic activity was assessed by combining 100 L of supernatant (the enzyme extraction solution) with 2.9 mL of H_2_O_2_ (20 mmol L^-1^), and then absorbance was measured at 240 nm. The CAT activity was calculated in U kg^-1^. SOD’s enzymatic reaction system included 1.7 mL of 50 mmol L^-1^ phosphate buffer (pH = 7.8), the reaction mixture (each 0.3 mL of 30 mmol L^-1^ methionine (MET), 750 μmol L^-1^ nitrotetrazolium blue chloride (NBT),100 μmol L^-1^ ethylenediaminetetraacetic acid disodium salt (EDTA-Na_2_), 0.3 mL of 20 μmol L^-1^ riboflavin, and 0.1 mL of enzyme extraction. Another reaction was carried out as the control (without the enzyme extraction solution) and was left in the dark. The reaction mixture containing the enzyme extraction supernatant was placed under 4000 lx fluorescent lamp. After 15 min reaction, the absorbance value of each tube was measured at 560 nm. The SOD activity was calculated in U kg^-1^.

#### POD and PPO enzymes activities

In order to extract POD and PPO, 5 g of freeze-dried grape berry powder was weighed. Then 5 mL of extraction buffer (1 mmol L^-1^ polyethylene glycol (PEG), 4% PVPP, 1% Trition X-100, acetic acid-sodium acetate buffer, pH = 5.5) was added. After being ground in an ice bath, the sample was centrifuged at 4°C for 30 min. at 12,000 × g. Then use the collected supernatant as the solution for extracting the POD and PPO enzyme activity. For POD detection, 0.5 mL of enzyme supernatant was added to 3 mL guaiacol (25 mmol L^-1^), which was then heated until the color dissipated and cooled to room temperature. After adding 200 L of H_2_O_2_ (0.5 mol L^-1^), the POD activity was measured at 470 nm and expressed in U kg^-1^. PPO activity was determined using 4 mL of acetic acid-sodium acetate buffer (pH = 5.5), 1 mL of 50 mmol L^-1^ catechol, and 100 L of enzyme extraction. When the reaction was finished, the absorbance of PPO was tested at 420 nm and reported as U kg^-1^.

### Sugars quantifications

#### Reducing sugar and soluble sugar levels

The quantification of reducing sugar and soluble sugar used the 3, 5-dinitrosalicylic acid method and anthrone colorimetry assay with a slight modification, respectively (Bonilla et al., 1999). The first step was to combine 1 g of freeze-dried berry powder with 25 mL of deionized water. Then, 1.5 mL of the 3, 5-dinitrosalicylic acid reagent was added, and the absorbance value was measured at 530 nm. For soluble solids, 50 mg of freeze-dried berry powder and 4 mL of 80% ethanol were combined. After 10 minutes of heating in a boiling water bath with 5 mL of the anthrone reagent, the absorbance value at 630 nm was recorded. Finally, the absorbance of reducing sugar and soluble solids was converted to glucose equivalents using the calibration curve drawn with glucose.

#### Fructose and glucose levels

The fructose and glucose from grape berries were extracted using the Nowicka et al. (2019) and Li et al. (2021a) protocols. In order to defrost, the freeze-dried grape powder that had been kept at -80°C was moved to 4°C. The grape juice was extracted after being centrifuged at 10,000 × g for 15 minutes. Then, 1 mL of grape juice was diluted with 9 mL of ultrapure water, and impurities were removed by filtering through a 0.22 m membrane.

The quantification of fructose and glucose was conducted on LC3000 Semi-preparation Isocratic HPLC System (CXTH, BJ, CHN), equipped with differential detector (CXTH, BJ, CHN) and Amino column (250 mm × 4.6 mm, 5 μm). There were three technical replicates carried out for each treatment and standard substance. All reagents were chromatographic grade and were purchased from Shanghai ANPEL Experimental Technology Co., Ltd (SHH, CHN).

### Fatty acids measurement

According to the procedure used in our lab (Li et al., 2021a), various FAs were extracted with minor adjustments. First, 1 g of freeze-dried berry powder was weighed, followed by 100 mg of pyrogallic acid, and various zeolites were added, along with 1 mL of glyceryl triundecanoate (internal standard, 5 g L^-1^). After that, 4 mL water and 2 mL ethanol (95%) were added and thoroughly mixed. After adding 2 mL of 2% sodium hydroxide methanol, the solution was heated in a water bath for 30 minutes at 85°C. Later, 3 mL of a 14% boron trifluoride methanol solution was added, and the mixture was once more heated in a water bath for 30 minutes at 85°C. After being cooled to room temperature (25°C), 1 mL of n-hexane was added to the solution and then allowed to react for 1 h. The solution was finally filtered through a 0.45 m membrane, and 100 μL of supernatant was collected to detect FAs.

In this study, FAs detection was carried out using a gas chromatograph (GC) instrument equipped with a TG-5MS column (30 mm × 0.25 mm × 0.25 μm). The oven temperature was initially set at 80°C for 1 min, then changed to 200°C at a rate of 10°C min^-1^, elevated to 250°C at 5°C min^-1^, and finally increased up to 270°C at 2°C min^-1^. In addition, the inlet temperature was 290°C, and the carrier gas flow rate was 1.2 mL min^-1^. All reagents were chromatographic grade and purchased from Shanghai ANPEL Experimental Technology Co., Ltd (SHH, CHN).

### Endogenous phytohormones determinations

Endogenous phytohormones extraction was performed using a well-established method with a little modification (Kojima et al., 2009) and identified by HPLC. Initially, 500 mg of freeze-dried grape powder was homogenized in 1 mL of the extracting solution (Methanol: ddH_2_O: Formic acid = 15:4:1), then the mixture was extracted for an overnight period at −20°C. The mixture was centrifuged for 20 minutes at 13,000 rpm and 4°C. The supernatant was evaporated using a rotary at 42°C after being filtered *via* a CNWBOND HC-C18 SPE Cartridge (CNW, German). The rotary residue was dissolved in 5 mL of 1 M formic acids, transferred immediately into a Poly-Sery MCX SPE Cartridge (CNW, German), and then eluted with 5 mL of 1 M formic acid and methanol. Finally, rotary drying was carried out once more, and the residue was redissolved in 0.5 mL of extraction solution (Methanol: Isopropanol: Acetic acid = 20:79:1), and the liquid containing GA_3_, indole propionic acid (IPA), SA, IAA, indolebutyric acid (IBA), JA, and MeJA was obtained. Then, the Poly-Sery MCX SPE Cartridge was successively eluted with 5 mL of 0.35 mol L^-1^ ammonia and 5 mL of 0.35 mol L^-1^ ammonia (in 60% methanol). The filtrate was then rotary evaporated at 60°C, and the residue was added to 0.5 mL of 5% acetonitrile. Afterward, a liquid containing the substances zeatin (ZT), zeatin riboside (ZR), N-6 isopentenyl adenine (ip), N-6 isopentenyl adenine nucleoside (ipR), and kinetin (KT) was attainable. Both liquids were utilized to identify endogenous phytohormones using an HPLC system (LC3000 Semi-preparation Isocratic), a UV-detector (CXTH, BJ, CHN), and a Capecell PAK C18 column (4.6 mm 100 mm, 1.8 mm). Each treatment and reference component had a minimum of three technical replicates selected. The reagents used in this investigation were all chromatographic quality and obtained from Shanghai ANPEL Experimental Technology Co., Ltd (Shanghai, China).

BR was determined using the protocols and instructions of the commercial enzyme-linked immunosorbent assay (Elisa) kit manufactured by Shanghai Yanqi Biotechnology Co., Ltd (Shanghai, China).

### RNA extraction and real-time RNA-Seq analysis

Total RNA was extracted from grape berries using the RNA Prep Pure Plant Plus Kit (TaKaRa, Dalian, China). BIO-RADXR gel imaging analysis system (Bio-Rad, CA, USA) was used to detect the purity and integrality of RNA extracted. qRT-PCR was performed using the CFX Connect Real-Time PCR Detection System (Bio-Rad, CA, USA). The steps were as follows: 95°C for 20 s, followed by 95°C for 39 cycles (15 s), 55°C for 15 s, and finally, 60°C for 15 s. The 2^-△△Ct^ assay determined the genes’ relative expression. All genes and transcription factor sequences were acquired from EnsemblPlants (http://plants.ensembl.org/info/about/index.html). Furthermore, the primers for qRT-PCR were designed qPrimerDB-qPCR Primer Database (https://biodb.swu.edu.cn/qprimerdb/). All specific information of all sequences is presented in **Table 1**.

**Table 1:** Primers for qRT-PCR, all primers were designed by Primer Premier 5.0 and qPrimerDB-qPCR Primer Database.

Target genes	Forward sequence (5’ → 3’)	Reverse Sequence (5’ → 3’)
*VvPAL (VIT_11s0016g01520)*	CTCACACCACAACGGCAACG	CGCCACCATTCTCTTCACCTC
*VvC4H (VIT_06s0004g08150)*	CAATGGCAATGACTTCAGGTAC	GACGTCCAATTGTGATACCAAG
*Vv4CL (VIT_16s0039g02040)*	ACCACCTCCCTCTCCACAC	ACCACCTCCCTCTCCACAC
*VvCHS (VIT_14s0068g00930)*	AGCCAGTGAAGCAGGTAGCC	GTGATCCGGAAGTAGTAAT
*VvCHI (VIT_13s0067g03820)*	AGACTGTGGAGGAGTTAGCG	AGAATGGAGTTGCCTGGTG
*VvF3H (VIT_04s0023g03370)*	CCAATCATAGCAGACTGTCC	TCAGAGGATACACGGTTGCC
*VvF3'5'H (VIT_06s0009g02970)*	ACTAAGCCACAGGAAACTAA	AAACCGCTCAGACCAAAACC
*VvFLS (VIT_18s0001g03430)*	AAACCACCTACTTACAGAGC	ACCTAACCCCAGTGACAGAC
*VvDFR (VIT_18s0001g12800)*	GAAACCTGTAGATGGCAGGA	GGCCAAATCAAACTACCAGA
*VvLDOX (VIT_02s0025g04720)*	ACTCTTTGGGGATTGACTGG	AGGGAAGGGAAAACAAGTAG
*VvLAR (VIT_01s0011g02960)*	TGCTTTTGTGATTTTGTTAGAGG	CCCTTCCCCGATTGAGAGTA
*Vv3GT (VIT_14s0006g03000)*	GGGATGGTAATGGCTGTGG	ACATGGGTGGAGAGTGAGTT
*Vv5GT (VIT_15s0021g00910)*	TTCCATGGCTGAACTCAAAAC	AACATCCAACTGCTTGGTGAC
*VvANR (VIT_00s0361g00040)*	CAATACCAGTGTTCCTGAGC	AAACTGAACCCCTCTTTCAC
*Vvactin*	GACAATTTCCCGTTCAGCAGT	GATTCTGGTGATGGTGTGAGT
*VvPDH (VIT_09s0002g07930)*	AATTACAGAGGCTGGCTTTACT	GGAAAACAACAGGATCAAGGTC
*VvPDC1 (VIT_13s0067g00340)*	GCAAGAAGGTGATGTTGTCATTGC	ACTTGAGAGATACCGTAGGTGTTG
*VvARO10 (VIT_03s0038g02120)*	TTGCCATGCAAGACCACTCAA	TGCTGCACCGTCACCTTCAA
*VvADH1 (VIT_18s0001g15410)*	GCTGGTGCCAAGTGTTGTTCT	GCTTCTCTGGTGTCAGCTCTGTT
*VvACC1 (VIT_12s0059g01380)*	AAGGTCGCTCTACAAGCAAGAG	TGAGCCATAGGCAACCTTCAC
*VvFAS1 (VIT_01s0011g06640)*	TGCTAGATTGGCCGGAAAGTACA	TCGATGATTTCCTTGATAGGTTCGG
*VvOLE1 (VIT_14s0066g00700)*	GGTATTGTTCACGACGTATCTGGTT	GCCTTGGTAGCGTCCTTACCT
*VvELO1 (VIT_04s0023g01140)*	GCAGTTCATGCTTGATCTCATTGTC	CAATCCTCACACTGTGGAGTACAAG
*VvIAH1 (VIT_10s0003g00960)*	CCGTACCAACGAGAACTTTGCC	GCCAAGCATCACCACCTTCC
*VvFAT (VIT_05s0094g00930)*	ATTGCCGCTGATCGCTAAGG	CGCTGACGCCGCTATTGAC
*VvACX2 (VIT_00s0662g00010)*	CAGAAAGGAACAAAAGGAGACG	CTCCATCTCGAACACGATCTAA
*VvPCK1 (VIT_07s0205g00070)*	AGGGCTTATCATTCACTGTTCA	GTAGAGGATGTCATGTAGTGGG
*VvMFP2 (VIT_05s0077g02140)*	CTTGCAAGTAACACTTCGACAA	AAAGAAATGGGCTCCAACAATC
*VvEEB1 (VIT_11s0016g01930)*	AGCCATGAGCCTCTCATCAGG	TGAGCAACCTCTAGCATTAAGGAC
*VvATF1 (VIT_16s0039g00570)*	GGACCGAGTTGGCGGCTAAT	GTGGATCGAAGACCGACCATCA
*VvFRK (VIT_01s0011g00240)*	GATCTAAATCTTCCGTTGCCAC	CAAGTTCTTGCTTGGTAACCTC
*VvTK (VIT_10s0003g03770)*	CATTTGCGATCTGAATTCGACT	GAAAGGAGAGGAAGTGATGTGA
*VvALS (VIT_16s0022g01030)*	TGCAGCATTACTCCAATTTTCC	CTTTGTGCTGGTAGGTTTATGG
*VvTPI (VIT_03s0038g01780)*	AAGACACATGGAATGAACAACG	CAATTCCCAACCCACATGTAAA
*VvPFK (VIT_14s0108g00540)*	CAAACCCTAAACCTCACGAATC	GTTAGAAAACCAACGGACCAAA
*VvCSY (VIT_12s0142g00610)*	GTGATGCGGGGATTCTTAGATA	TCCCATACATTAAGAGGTACGC
*VvDNL (VIT_13s0019g04580)*	AATTACAGAGGCTGGCTTTACT	GGAAAACAACAGGATCAAGGTC
*VvSUS (VIT_11s0016g00470)*	CAGCAGCAACAGCAATAACTAA	GCAGATGCACACACTTTATTGA
*VvINV (VIT_06s0061g01520)*	CCAGAAAACTTGTAGAAGCACC	GTTGACGCATTCCTTAAGGATC
*VvGPDH (VIT_14s0219g00280)*	TTGTCAAAGGGTATAGAGGCTG	TGTTTTCCATAGCTACACCAGT
*VvPGK (VIT_19s0085g00380)*	AATACTTGAAACCTTCGGTTGC	CAATTTTCGATGACACCTTCGA
*VvENO (VIT_17s0000g04540)*	CTCAGAGCCTTTGTGAACTCTA	TGCTTCAGCTATTCTCTTTGGA
*VvGPI (VIT_18s0001g07280)*	GACATTCATTGAAGTTCTGCGT	ATATTCCAACTGCTCGCTCATA
*VvGI (VIT_06s0004g06020)*	TTCAAGCAAAAGATCACGAAGG	CAGAGACGAAGGCTTATAGTGT
*VvPK (VIT_08s0007g04170)*	GATCTAAATCTTCCGTTGCCAC	CAAGTTCTTGCTTGGTAACCTC
*VvHT1 (VIT_05s0020g03140)*	CACTTTTGTCATAGCACAGTCC	ATTGGAATGTTTTTGGTCTCCG
*VvHXK (VIT_18s0001g14230)*	GCTGAAAGCTTAATTCCTGGTT	TTCTTGGGCCATCTTTAGTAGG
*VvPEPCase (VIT_19s0014g01390)*	GGGATGTTTGCTTATTGGCTAG	CGTTCACGTGTTTGATACAGTT
*VvDWF4 (VIT_04s0023g01630)*	CTGAAGCATTCCAACCTTTCAA	GCCTTCCAAGAAGTAGATAGCT
*VvBR6OX1 (VIT_14s0083g01110)*	TTCCATGATGGCTGTCAAGTAT	CGCATCAACTTGTAGTCATTCC
*VvBR6OX2 (VIT_01s0011g00190)*	CAGAAAGATTGATCAGGGCATG	GGCTGATTCAGTTTCCATGATC
*VvBAS1 (VIT_18s0001g12200)*	ATTGCAGCATGACATACTTCAC	CAAGGGGTTAAACCTAGCCTTA
*VvDET2 (VIT_08s0007g01760)*	TCTGTACACATGTGCCAATTTG	TGGTACATCGTCATTCACTTGA
*VvCPD (VIT_13s0067g00660)*	GAAGAGGAGAAGAAGAACGACA	TTATCCTTGCCCTAATCTCGTC
*VvROT3 (VIT_04s0023g02650)*	GATTATGCCTGGACTGACTACA	TTAACAGCTTTTCTCCAAACCG
*VvLipase (VIT_12s0059g01550)*	ATTTGTAATAAACCGGGCATCG	GGGATGTTCGTCATGTTGTATG
*VvLOX (VIT_14s0128g00780)*	ACAACTTCCACGAAAACTTACG	GAGTCATTCACAGCAGCATAAG
*VvAOS (VIT_03s0063g01850)*	AAGTCGAGAAGAGAAACGTCTT	GTAGAGAGAAGGAGAAGCGTTT
*VvAOC (VIT_14s0083g00110)*	GCCACATACAGCTTCTATTTCG	AGATACGTATCCTCGTAGGTCA
*Vvopr3 (VIT_11s0016g01230)*	TCAAACCGGTGCAAATTCTTAG	GTGATCAATTGCTGGTGAAACT
*Vvopcl1 (VIT_01s0010g03720)*	TGTGCACCAGAATGATCTATGT	ATGTGAAAGAATGGCATTAGGC
*Vvacx1 (VIT_00s0662g00010)*	CTTCGCAAGCATTCTAAGACTC	GCAACATAATCTACAATGCGGT
*Vvacx5 (VIT_05s0020g01740)*	CGCTCCTTCGTCAAAGATTTAC	CCAAGATACCAGACTATGACCC
*VvJMT (VIT_18s0001g12900)*	GAGCTGGAACAAGGGAAAAATT	CACTATTTCTTCTGCACGTGAC
*VvMJE1*	ATGGAGAAAAGAGAGAGGCA	CTCTCCATAGCAACCGATACG
*VvJAR1 (VIT_15s0046g01280)*	GAATCTTCTGCTGACGATCAAC	CCAGGATCAGTCGATTTATCCA
*VvJIH1 (VIT_18s0001g02570)*	CGGACTGCACAATTTGGATTAA	GGTATTGAATTCCTCGAAAGCC

### Statistical analysis and figure drawing

Results analysis was performed using the IBM SPSS 16.0 statistical software package (Armonk, NY, USA). At least three biological replications were chosen for each treatment, and a univariate ANOVA test was used to analyze variance homogeneity in the form of mean ± standard error (SE). Pearson correlation analysis used to evaluate the fluctuation trends between phytohormones and quality-related biochemical traits such as antioxidant activity and fatty acid content were examined through SPSS 16.0 (IBM, Armonk, NY, USA). All figures were constructed using GraphPad Prism 9.0 (GraphPad Software Inc., San Diego, CA, USA), TBtools (CAN, CHN), and Visio 2020 (Microsoft, SEA, USA). The HPLC’s detection and quantification limits are listed in **Table S1**.

## Results and discussion

### Physiological parameters

**Figure 1:**
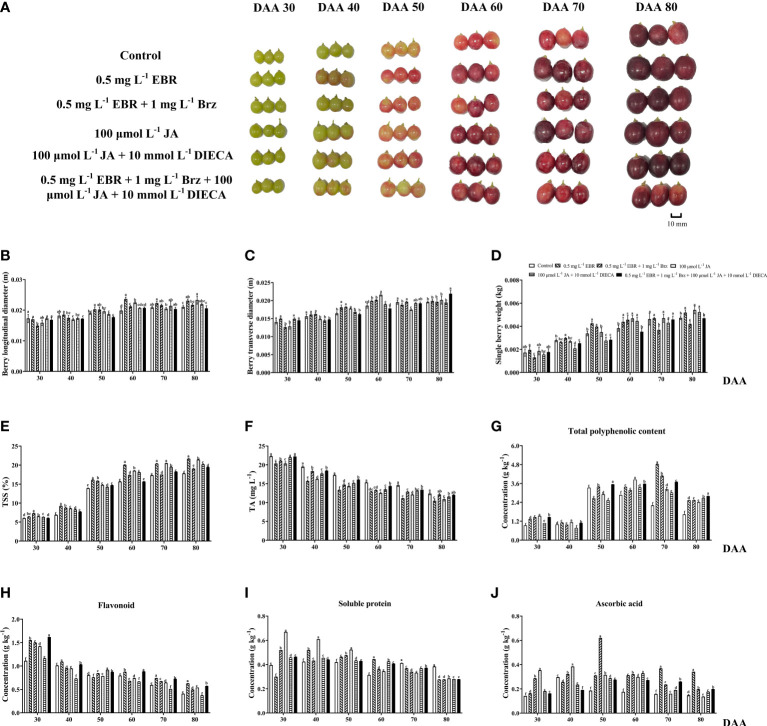
Grape berry phenotypes and berry quality-related parameters. **(A)** Grape berries were collected in six treatment groups (Control, 0.5 mg L^-1^ EBR, 100 μmol L^-1^ JA, 0.5 mg L^-1^ EBR + 1 mg L^-1^ Brz, 100 μmol L^-1^ JA + 10 mmol L^-1^ DIECA, 0.5 mg L^-1^ EBR + 1 mg L^-1^ Brz + 100 μmol L^-1^ JA + 10 mmol L^-1^ DIECA) at six sampling stages (DAA 30, 40, 50, 60, 70, 80). **(B-J)** Effect of exogenous EBR, JA and their signaling inhibitors on grape berry quality in different treatment groups (Control, 0.5 mg L^-1^ EBR, 100 μmol L^-1^ JA, 0.5 mg L^-1^ EBR + 1 mg L^-1^ Brz, 100 μmol L^-1^ JA + 10 mmol L^-1^ DIECA, 0.5 mg L^-1^ EBR + 1 mg L^-1^ Brz + 100 μmol L^-1^ JA + 10 mmol L^-1^ DIECA) at six sampling stages (DAA 30, 40, 50, 60, 70, 80). Parameters including berry longitudinal diameters, berry transverse diameters, single berry weight, TSS, TA, total phenolic contents, flavonoid contents, soluble protein contents, and ascorbic acid contents were shown in histograms. Error bars = ± SE (n≥3). Significant differences among samples have been displayed by a–f letters (one-way ANOVA, P<0.05).

Physiological parameters were measured in EBR, JA, and their signaling inhibitors (Brz and DIECA) treatment groups throughout the fruit’s development and ripening stages (**Figures 1B–D**). These parameters comprised longitudinal diameter, transverse diameter, and single-berry weight. Among treatments, the exogenous application of EBR and JA significantly contributed to fruit enlargement, especially during the late fruit ripening stage. The EBR and JA were higher than the control group in terms of longitudinal diameter (18.99% and 13.09%, respectively) at DAA 60, transverse diameter (10.10% at DAA 50 and 16.43% at DAA 60, respectively), and single berry weight (26% at DAA 50 and 22.01% at DAA 60, respectively).

### Biochemical properties

During the ripening of grapes, the TSS content was consistently increased while titratable acid (TA) decreased (**Figures 1E, F**). Exogenous EBR and JA application enhanced TSS contents over control treatment while reducing TA contents throughout the study. It was consistent with the work that exogenous EBR (Ghorbani et al., 2017) and JA (García-Pastor et al., 2019) considerably increase TSS and decrease TA in the grapevine.

Additionally, the TPC in EBR, JA, and control increased gradually from DAA 30–70, DAA 30–60, and DAA 30–50, respectively, then decreased. TPC was significantly higher in the exogenous EBR and JA treatment groups compared to the control group, despite DAA 50 (**Figure 1G**). Nevertheless, TFC continuously declined as grapes matured and ripened. Exogenous EBR and JA-treated grapes had considerably higher TFC activity than the control group. At DAA 30, 70, and 80, the EBR increased the TFA more than the control by 40.07%, 24.41%, and 53.61%, and the JA by 28.25%, 8.56%, and 32.42%, respectively (**Figure 1H**). In terms of soluble protein, the results indicated an increasing trend at the beginning of berry growth; all six treatment groups attained their peak at DAA 30–50. When given exogenous EBR and JA treatments, soluble protein contents at DAA 40, DAA 50, and DAA 60 were significantly improved (**Figure 1I**). Ascorbic acid’s variation pattern was almost identical to that of TFC, and all applications revealed an upward trend from DAA 40 to 70. Except for DAA 40 and 80, exogenous EBR and JA treatments significantly influenced ascorbic acid contents throughout the research (**Figure 1J**). According to the previous study that supported our findings, TAC, TFC, and TPC were reported to be significantly enhanced in ‘Cabernet Sauvignon’ and ‘Yan 73’ grape berries after applying 0.4 mg L^-1^ of EBR (Xi et al., 2013) and 100 μmol L^-1^ of MeJA, respectively (Wang et al., 2019). In EBR and JA, the addition of Brz and DIECA causes a decrease in the weight of the berries and the concentrations of soluble protein at DAA 70. Compared to the control group, the berry weight and soluble protein dropped by 20.03% and 17.45% in the EBR+Brz treatment, 6.72% and 10.54% in the JA+DIECA treatment, and 0.55% and 9.6% in the EBR+Brz+JA+DIECA treatment, respectively.

Exogenous EBR increased TSS content in carambola fruit (Zhu et al., 2021), and JA stimulated ripening, anthocyanins formation, and the volatilization of hexanol, linalool, and α-terpineol in peach fruit (Wei et al., 2017). Additionally, exogenous EBR treatment increased the activity of several lipid peroxidation and defense-related enzymes, which maintained the firmness of grape berries at a greater level and reduced the rate of fruit drop and decay (Liu et al., 2016). In this study, we verified that exogenous EBR and JA effectively promote berry enlargement, sugar accumulation, and the formation of secondary metabolites (phenolic, soluble protein, flavonoids, and ascorbic acid).

### Antioxidant enzymes activity

SOD, POD, CAT, and PPO are the main metabolites reflecting antioxidant activity in plants; Pro and MDA could reflect the degree of free radical accumulation and cell membrane damage (Yang et al., 2021b). **Figures S2A–C** showed that the variation trends of antioxidant enzymes, including CAT, POD, and SOD were similar; firstly, they increased and subsequently slightly decreased with the ripening of grapes. Throughout the whole phase of berry development, exogenous EBR and JA raised CAT and POD contents, whereas the contents of SOD only noticeably rose at DAA 70 and DAA 80. Additionally, PPO, MDA, and Pro variation trends remained escalating. The maximum PPO activity was noted at DAA 80, and the EBR and JA treatments were superior to the control group by 42.11% and 61.92%, respectively at DAA 60. In terms of Pro activity, the application of exogenous EBR resulted in increases at DAAs 60, 70, and 80 of 53.65%, 45.67%, and 311.46%, respectively, whereas the JA treatment increases at these same DAAs of 108.73%, 247.19%, and 51.61%. Interestingly, at DAA 30, 40 and 70, exogenous EBR and JA had an inhibiting impact on MDA formation (**Figures S2D–F**). Our results further supported the findings of Pakkish et al. (2019), who discovered that EBR application significantly elevated the activities of CAT and POD in ‘Rish Baba’ grapes, and the outcome of Yosefi and Javadi (2020), that exogenous JA increased the accumulation of SOD and POD for improving water stress tolerance *in vitro* strawberry plants. Wang et al. (2019) observed that MeJA treatment suppressed MDA accumulation in blueberry fruits, and EBR treatment promoted Pro accumulation to restore the damaged phenotype of peach fruits caused by chilling injury (Gao et al., 2016). Also, exogenous MeJA significantly improved the post-harvest pear fruits’ resistance against blue mold disease by increasing PPO activity (Chen et al., 2022a). Additionally, the EBR+Brz treatment significantly reduced the accumulation of CAT, PPO, and MDA at DAA 60 by 19.88%, 15.78%, and 67.81% than the control group, respectively. The generation of POD and SOD was lower in the JA+DIECA group than in the control group at DAA 30, with differences of 21.28% and 11.63%, respectively. Regarding Pro activity, using Brz and DIECA dramatically reduced the biosynthesis at DAA 40. The pro content was lower in the treatments of EBR+Brz, JA+DIECA, and EBR+Brz+JA+DIECA than in the control, with values of 36.92%, 43.14%, and 6.81%, respectively.

In all, exogenous EBR and JA in this study were found to enhance enzymatic activity and reduce membrane lipid peroxidation in grape berries. MDA contents rose with the continuing development and ripening of grapes. Interestingly, CAT accumulated primarily at the veraison stage, whereas POD, SOD, PPO, and osmotic regulating substance (Pro) increased at maturity. Eventually, JA and EBR contributed to the improvement of antioxidant capacity in the grapevine.

### Anthocyanins biosynthesis and metabolism

**Figure 2:**
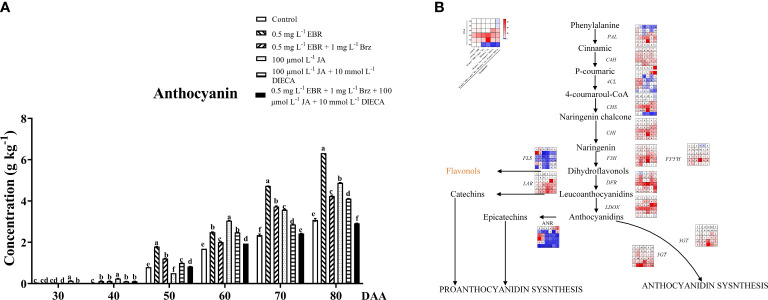
Effect of exogenous EBR, JA and their signaling inhibitors on anthocyanins accumulation. **(A)** Effect of exogenous EBR, JA and their signaling inhibitors on anthocyanins contents in different treatment groups (Control, 0.5 mg L^-1^ EBR, 100 μmol L^-1^ JA, 0.5 mg L^-1^ EBR + 1 mg L^-1^ Brz, 100 μmol L^-1^ JA + 10 mmol L^-1^ DIECA, 0.5 mg L^-1^ EBR + 1 mg L^-1^ Brz + 100 μmol L^-1^ JA + 10 mmol L^-1^ DIECA) at six sampling stages (DAA 30, 40, 50, 60, 70, 80). The levels of anthocyanins were expressed in the form of a histogram. Error bars = ± SE (n≥3). Significant differences among samples have been displayed by a–f letters (one-way ANOVA, P<0.05). **(B)** Expression of genes involved in anthocyanins biosynthesis and metabolic pathways in different treatment groups (Control, 0.5 mg L^-1^ EBR, 100 μmol L^-1^ JA, 0.5 mg L^-1^ EBR + 1 mg L^-1^ Brz, 100 μmol L^-1^ JA + 10 mmol L^-1^ DIECA, 0.5 mg L^-1^ EBR + 1 mg L^-1^ Brz + 100 μmol L^-1^ JA + 10 mmol L^-1^ DIECA) at six sampling stages (DAA 30, 40, 50, 60, 70, 80). The expression levels of genes were remarked as a heatmap. Significant differences among samples have been displayed by a–f letters (one-way ANOVA, P<0.05).

Anthocyanins, a type of natural pigment that widely exists in the cell fluid of plant roots, stems, leaves, fruit, and other organs, have many health functions in humans and are widely studied in grapevine (Mattioli et al., 2020). With fruit development and ripening, anthocyanins contents indicated an increasing trend in all treatment groups (**Figure 2A**). After the veraison, the color of the grape berry changed to pink and darkened as it developed and ripened (**Figure 1A**). Similar results were seen in other research, where exogenous 24-EBR interacted with light to regulate the accumulation of anthocyanins and proanthocyanidins in ‘Cabernet sauvignon’ (Zhou et al., 2018), as well as markedly increased total anthocyanins biosynthesis to improve the coloring of peach fruits (Wei et al., 2017).

According to a transcription regulation study, as shown in **Figure 2B**, the expression levels of *PAL* (*VIT_11s0016g01520*), *CHI* (*VIT_13s0067g03820*), *F3’5’H* (*VIT_06s0009g02970*), *LDOX* (*VIT_02s0025g04720*), and *LAR* (*VIT_01s0011g02960*) were higher under exogenous EBR and JA treatments than the control group at DAA 60 and DAA 70. Notably, exogenous EBR and JA treatments increased the expression of downstream genes directly involved in the production of anthocyanins, such as *3GT* (*VIT_14s0006g03000*) and *5GT* (*VIT_15s0021g00910*), except for DAA 40 and DAA 70. Similarly, the exogenous EBR and JA treatments at DAA 50 and DAA 70 resulted in decreased expression levels of *FLS* (*VIT_18s0001g03430*; dihydroflavonols to flavonols), *ANR* (*VIT_00s0361g00040*; anthocyanidins to Epicatechins), and *4CL* (*VIT_16s0039g02040*; anthocyanidins biosynthesis) than the control. In line with earlier research, Zhou et al. (2018) discovered that exogenous EBR developed the pericarp color of ‘Cabernet Sauvignon’ grapes by up-regulating the expression of anthocyanin-related genes (*VvCHI1*, *VvCHS3*, *VvF3’5’H*, *VvDFR*, and *VvUFGT*). Additionally, MeJA also significantly increased the expression of anthocyanin biosynthesis-related genes (*PpCHS*, *PpCHI*, *PpF3H*, *PpDFR*, *PpUFGT*), as well as a transcription factor (PpMYB), which was the primary consequence of the significant anthocyanin accumulation in the peach pericarp (Wei et al., 2017). Moreover, we also found Brz and DIECA could not only significantly reduce the content of total anthocyanins at DAA 60–80 but also inhibit the up-regulation effect of exogenous EBR and JA on downstream genes of anthocyanin and proanthocyanin biosynthesis (*DFR*, *LDOX*, *3GT*, *5GT*, *ANR*, *LAR*) at different developmental stages.

Our findings provided evidence that exogenous EBR and JA played an important role in promoting the accumulation of anthocyanins in grape berries, reflected by the huge increase in its content during fruit ripening and the up-regulation of gene expressions related to its biosynthesis.

### Sugars biosynthesis, metabolism and transportation

**Figure 3:**
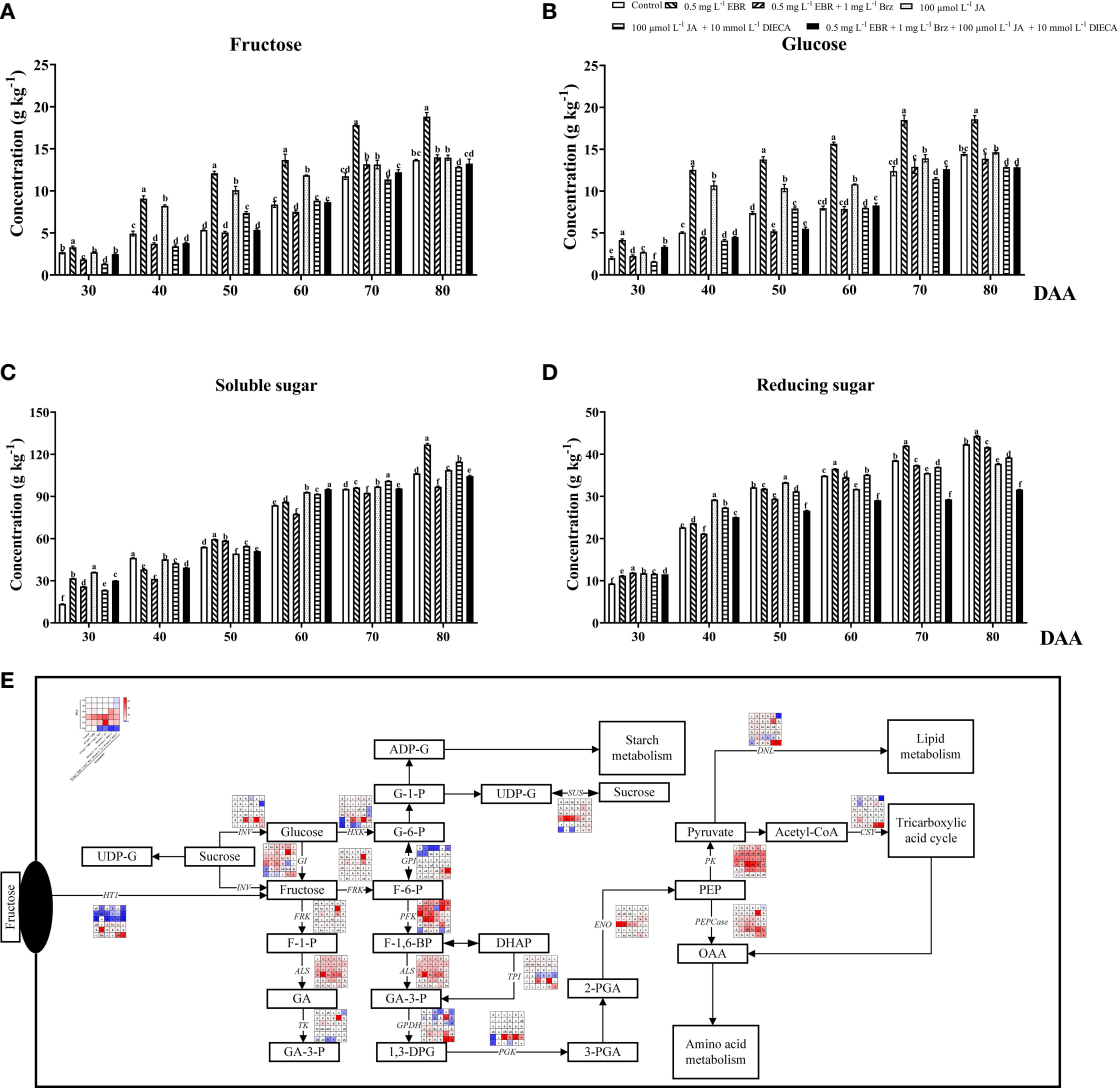
Effect of exogenous EBR, JA and their signaling inhibitors on sugars formation and transportation. **(A-D)** Effect of exogenous EBR, JA and their signaling inhibitors on different types of sugar content in different treatment groups (Control, 0.5 mg L^-1^ EBR, 100 μmol L^-1^ JA, 0.5 mg L^-1^ EBR + 1 mg L^-1^ Brz, 100 μmol L^-1^ JA + 10 mmol L^-1^ DIECA, 0.5 mg L^-1^ EBR + 1 mg L^-1^ Brz + 100 μmol L^-1^ JA + 10 mmol L^-1^ DIECA) at six sampling stages (DAA 30, 40, 50, 60, 70, 80). **(A)** Fructose contents. **(B)** Glucose contents. **(C)** Soluble sugar contents. **(D)** Reducing sugar contents. The levels of each sugar were expressed in the form of a histogram. Error bars = ± SE (n≥3). Significant differences among samples have been displayed by a–f letters (one-way ANOVA, P<0.05). **(E)** Expression of genes involved in sugars biosynthesis, metabolic and transporting pathways in different treatment groups (Control, 0.5 mg L^-1^ EBR, 100 μmol L^-1^ JA, 0.5 mg L^-1^ EBR + 1 mg L^-1^ Brz, 100 μmol L^-1^ JA + 10 mmol L^-1^ DIECA, 0.5 mg L^-1^ EBR + 1 mg L^-1^ Brz + 100 μmol L^-1^ JA + 10 mmol L^-1^ DIECA) at six sampling stages (DAA 30, 40, 50, 60, 70, 80). The expression levels of genes were remarked as a heatmap. Significant differences among samples have been displayed by a–f letters (one-way ANOVA, P<0.05).

The main sugars found in grape berries are fructose and glucose (Trad et al., 2021). In addition to measuring fructose and glucose, this study quantified genes involved in the biosynthesis and metabolism of sugars using qRT-PCR (**Figure 3**). Exogenous EBR and JA application significantly elevated the accumulation of fructose and glucose contents, except at DAA 30 and DAA 80 (**Figures 3A, B**). Furthermore, the trend of soluble and reducing sugars increased with fruit ripening, similar to fructose and glucose (**Figures 3C, D**). Apart from DAA 40 and DAA 50, exogenous EBR treatment considerably enhanced the content of soluble and reducing sugars. Similarly, Zhu et al. (2021) found that exogenous application of 2,4-EBR treatment promoted the accumulation of sugars in carambola during the late fruit ripening stage. Nevertheless, under exogenous JA treatment, we observed that soluble sugar contents declined at DAA 40 and DAA 50 (a decrease of 2.37% and 8.74% than the control group, respectively) while reducing sugar contents decreased at DAA 60, 70, and 80 (a decline of 9.07%, 7.85% and 10.90% than the control group, respectively). Alike in previous studies, glucose and fructose content were significantly lower in MeJA-treated tomato fruit than in the control group during post-harvest ripening (Tao et al., 2021). Compared with EBR and JA treatments, Brz and DIECA effectively inhibited sugar formation at DAA 40 to 80 and DAA 30 to 40, respectively. Additionally, the combined action of these two signaling inhibitors demonstrated a potent decreasing effect on both soluble and reducing sugar.

As **Figure 3E** demonstrated that exogenous EBR and JA treatments elevated the expression of genes involved in fructose metabolism, including *ALS* (*VIT_03s0038g00670*), *FRK* (*VIT_01s0011g00240*), *TK* (*VIT_10s0003g03770*) and *GI* (*VIT_06s0004g06020*), compared to the control group at DAA 70. Exogenous EBR and JA treatments at DAA 50 and 70 up-regulated the expression of the genes involved in fructose accumulation (*INV; VIT_06s0061g01520*) and transportation (*HT1*; *VIT_05s0020g03140*). Further investigation on the biosynthesis of phosphoenolpyruvate (PEP) revealed that exogenous EBR and JA treatments significantly up-regulated the expression of *PGK* (*VIT_19s0085g00380*) and *TPI* (*VIT_03s0038g01780*) at DAA 30 and 40, as well as *GPDH* (*VIT_14s0219g00280*) and *ENO* (*VIT_17s0000g04540*) at DAA 80. Regarding glucose metabolism, similar variation trends of *HXK (VIT_18s0001g14230)* and *SUS (VIT_11s0016g00470)* were seen among treatments. Exogenous EBR and JA application elevated their glucose metabolism gene expressions at DAA 30, DAA 70, and DAA 80 compared to the control group. Additionally, when exogenous EBR and JA treatments were applied to grape berries, the expression levels of the genes involved in lipid metabolism (*DNL*, *VIT_13s0019g04580* and *PK*, *VIT_08s0007g04170* at DAA 30 and 80), amino acid metabolism (*PEPCase*, *VIT_19s0014g01390* at DAA 50 to 80), and tricarboxylic acid regulation (*CSY*, *VIT_12s0142g00610* at DAA 80) were significantly higher. Hu et al. (2022) also discovered that the PpGATA12 gene was greatly influenced by exogenous EBR and regulated peach fruit’s energy and sucrose metabolisms. And exogenous MeJA increased soluble solids in tomato fruit by activating sucrose phosphate synthase (SPS) and sucrose synthase (SUS) activities (Li et al., 2021b). It should be noteworthy that adding Brz and DIECA increased the expression of some important genes for sugar biosynthesis, metabolism, and transportation (*HT1*, *GI*, *HXK*, *SUS*, *FRK*, *PFK*) at DAA 30, 70, and 80. For this reason, we concluded that the inhibition effect of signal inhibitors on sugar accumulation was likely caused by restrained sugar signaling transduction. Similarly, Brz addition downregulated the expression of *VvHT1* and *VvSUC27*, while EBR treatment enhanced the transcription levels of hexose transporters (*VvHT3*, *VvHT4*, *VvHT5*, and *VvHT6*) and sugar transporters (*VvSUC27*) on ‘Cabernet Sauvignon’ grape berries (Xu et al., 2015).

According to our findings, we came to the conclusion that the formation of sugars was extremely sensitive to BR and JA signals and that exogenous EBR and JA enhance the expression of genes involved in the biosynthesis, metabolism, and transport of sugars, ultimately leading to a significant improvement in grape berries’ flavor. Although the application of Brz and DIECA increased the expression of some important structural genes for sugars biosynthesis, metabolism, and transportation (*HT1*, *GI*, *HXK*, *SUS*, *FRK*, *PFK*), the total quantity of sugars decreased. As a result, we conclude that Brz and DIECA may restrict the accumulation of sugars by suppressing the expression of transcription factors associated with sugar signaling rather than directly influencing the expression of structural genes involved in the production or metabolism of sugars.

### Fatty acids biosynthesis and metabolism

**Figure 4:**
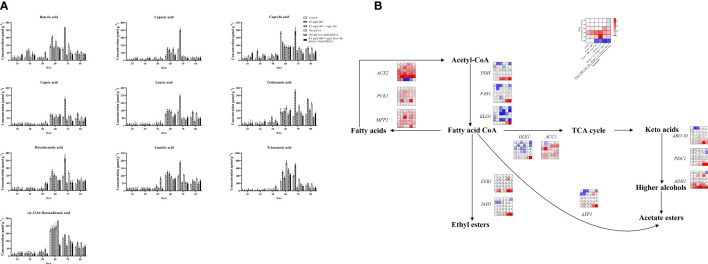
Effect of exogenous EBR, JA and their signaling inhibitors on fatty acids enrichment. **(A)** Effect of exogenous EBR, JA and their signaling inhibitors on diverse fatty acids contents in different treatment groups (Control, 0.5 mg L^-1^ EBR, 100 μmol L^-1^ JA, 0.5 mg L^-1^ EBR + 1 mg L^-1^ Brz, 100 μmol L^-1^ JA + 10 mmol L^-1^ DIECA, 0.5 mg L^-1^ EBR + 1 mg L^-1^ Brz + 100 μmol L^-1^ JA + 10 mmol L^-1^ DIECA) at six sampling stages (DAA 30, 40, 50, 60, 70, 80). A total of ten kinds of fatty acids were identified using GC, the levels of each fatty acid were expressed as a heatmap, red indicated that the fatty acid had a higher content, and blue indicated that the fatty acid had a lower content. Significant differences among samples have been displayed by a–f letters (one-way ANOVA, P<0.05). **(B)** Expression of genes involved in fatty acids biosynthesis and metabolic pathways in different treatment groups (Control, 0.5 mg L^-1^ EBR, 100 μmol L^-1^ JA, 0.5 mg L^-1^ EBR + 1 mg L^-1^ Brz, 100 μmol L^-1^ JA + 10 mmol L^-1^ DIECA, 0.5 mg L^-1^ EBR + 1 mg L^-1^ Brz + 100 μmol L^-1^ JA + 10 mmol L^-1^ DIECA) at six sampling stages (DAA 30, 40, 50, 60, 70, 80). The expression levels of genes were remarked as a heatmap. Significant differences among samples have been displayed by a–f letters (one-way ANOVA, P<0.05).

Fatty acids are different substances that accurately represent fruit flavor and grape berry quality (Hu et al., 2015). In this study, the abundance of butyric acid, caproic acid, caprylic acid, capric acid, lauric acid, tridecanoic acid, hexadecanoic acid, linoleic acid, tricosanoic acid, cis-13,16-Docosadienoic acid in grape berries were all accumulated at maturity stages (DAA 60, DAA 70 and DAA 80) (**Figure 4A**). Exogenous EBR and JA accelerated the accumulations of butyric acid, caprylic acid, capric acid, tridecanoic acid, hexadecanoic acid, linoleic acid, and cis-13,16-Docosadienoic acid at DAA 70 and DAA 80 compared to the control group. After applying Brz and DIECA, the contents of all fatty acids were decreased. Our results helped to clarify the functions of exogenous EBR and JA in the changes of fatty acids in grape berries, just as earlier research had shown that BR enhanced the level of unsaturated fatty acids in banana fruit and improved its capability to resist at low temperatures (Zhang et al., 2022). As well, MeJA also induced the biosynthesis of several fatty acids (especially oleic, linoleic acid, and linolenic acids) in tomatoes, which led to a significant improvement in the flavor of the tomato (Meza et al., 2022).

Furthermore, EBR treatment increased the loquat’s defense to withstand cold temperatures by decreasing the reduction of oleic, linoleic, and linolenic acids as well as the activities of lipoxygenase (LOX) and phospholipase D (PLD) at the late fruit ripening stage (Chen et al., 2022b). Exogenous MeJA has been shown to alter the amounts of fatty acids in phospholipids by activating the JA-mediated C-repeat-binding factor pathway, thereby reducing the chilling injury in peach fruits (Chen et al., 2019). Specific stress resistance, such as frost resistance, drought resistance, heat resistance, etc., will also be impacted by changes in the amount of various fatty acid monomers. In this study, the expression profiles of genes involved in fatty acids biosynthesis and metabolic pathways were studied to understand better the mechanism of exogenous EBR and JA in regulating fatty acids in grape berries (**Figure 4B**). We discovered that exogenous EBR and JA treatments increased the expression of genes involved in fatty acid metabolism at DAA 30, 40, and 70, such as *ACX2 (VIT_00s0662g00010)*, *PCK1 (VIT_07s0205g00070)*, and *MFP2 (VIT_05s0077g02140)*, as well as genes involved in the synthesis of fatty acids CoA at DAA 80, including *PDH* (*VIT_09s0002g07930*)*, FAS1* (*VIT_01s0011g06640*), and *ELO1* (*VIT_04s0023g01140*). Under exogenous EBR and JA treatments, genes responsible for fatty acids CoA metabolism and controlled TCA cycle of *OLE1 (VIT_14s0066g00700)* and *ACC1 (VIT_12s0059g01380)*; *ARO10 (VIT_03s0038g02120)*, *PDC1 (VIT_13s0067g00340)*, and *ADH1 (VIT_18s0001g15410)* regulated higher alcohols biosynthesis; *ATF1 (VIT_16s0039g00570)* regulated acetate esters biosynthesis; *EEB1 (VIT_11s0016g01930)* and *IAH1 (VIT_10s0003g00960)* regulated ethyl esters biosynthesis were all expressed upregulated trend at DAA 70. Following Brz and DIECA treatments, important genes for FAs biosynthesis (*PDH*, *FAS1*; DAA 60 and 70) were down-regulated, while genes for FAs metabolism (*ACX2*, *PCK1*, *MFP2*; DAA 30 and 40) were up-regulated. Subsequently, the concentration of fatty acids in grapes was significantly decreased.

Our study’s findings helped us better understand the regulatory mechanism of exogenous EBR and JA on fatty acid biosynthesis. Since fatty acid production was extremely sensitive to BR and JA signals, BR and JA signaling inhibitors suppressed the stimulation of exogenous EBR and JA on the FAs accumulation in grape berries.

### BR biosynthesis and metabolism

**Figure 5:**
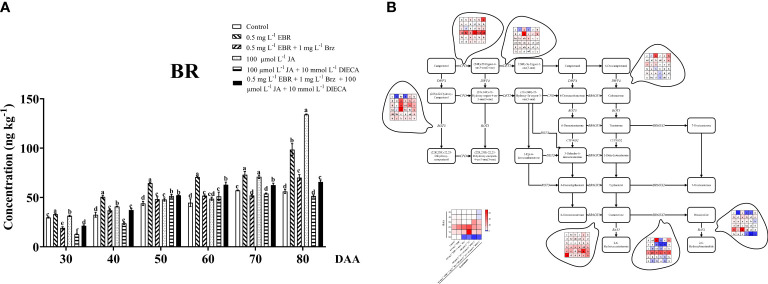
Effect of exogenous EBR, JA and their signaling inhibitors on BR biosynthesis and metabolism. **(A)** Effect of exogenous EBR, JA and their signaling inhibitors on BR contents in different treatment groups (Control, 0.5 mg L^-1^ EBR, 100 μmol L^-1^ JA, 0.5 mg L^-1^ EBR + 1 mg L^-1^ Brz, 100 μmol L^-1^ JA + 10 mmol L^-1^ DIECA, 0.5 mg L^-1^ EBR + 1 mg L^-1^ Brz + 100 μmol L^-1^ JA + 10 mmol L^-1^ DIECA) at six sampling stages (DAA 30, 40, 50, 60, 70, 80). The levels of BR were expressed in the form of a histogram. Error bars = ± SE (n≥3). Significant differences among samples have been displayed by a–f letters (one-way ANOVA, P<0.05). **(B)** Expression of genes involved in BR biosynthesis and metabolic pathways in different treatment groups (Control, 0.5 mg L^-1^ EBR, 100 μmol L^-1^ JA, 0.5 mg L^-1^ EBR + 1 mg L^-1^ Brz, 100 μmol L^-1^ JA + 10 mmol L^-1^ DIECA, 0.5 mg L^-1^ EBR + 1 mg L^-1^ Brz + 100 μmol L^-1^ JA + 10 mmol L^-1^ DIECA) at six sampling stages (DAA 30, 40, 50, 60, 70, 80). The expression levels of genes were remarked as heat map. Significant differences among samples have been displayed by a–f letters (one-way ANOVA, P<0.05).

Exogenous phytohormone application definitely influences the homeostasis of endogenous phytohormones in plants (Kosakivska et al., 2021). Further investigation was done on the BR content of grape berries in treatment. The amount of BR increased exponentially as grape berries continued to develop, and exogenous EBR and JA significantly helped in the continued development of endogenous BR (**Figure 5A**). In this study, we found that the levels of BR increased throughout the experiment, ranging from 111.5 to 175.5% in grape berries treated with exogenous EBR and from 105.9% to 238.9% in JA application compared to the control group.

It had been reported that EBR increased endogenous BR content, gene expression levels, and transcription factors related to BR signaling transduction (MdCYCD1, MdBAK1, MdBRI1, and MdBZR1) in apples (Mao et al., 2017). An extensive investigation of BR biosynthesis and metabolism at the transcription level was carried out to support the hypothesis that exogenous EBR and JA influenced endogenous BR biosynthesis (**Figure 5B**). The expression levels of the genes involved in BR biosynthesis, including *DWF4* (*VIT_04s0023g01630*), *BR6OX1* (*VIT_14s0083g01110*), *DET2* (*VIT_08s0007g01760*), and *ROT3* (*VIT_04s0023g02650*), increased as the fruit developed. Whereas the expression of the genes involved in BR biosynthesis (*BR6OX2*, *VIT_01s0011g00190*; *CPD*, *VIT_13s0067g00660*) and BR metabolism (*BAS1*, *VIT_18s0001g12200*) first increased and then reduced. Exogenous EBR and JA treatments reduced the expression of upstream BR biosynthesis genes (*DWF4*) compared to the control group at DAA 70 and DAA 80. Furthermore, the expression of *CPD* (the upstream gene in the BR biosynthesis pathway) was down-regulated at DAAs 40 and 50 and up-regulated at DAAs 60 and 80. Furthermore, exogenous EBR downregulated the expression of BR biosynthesis pathway genes *ROT3* (downstream gene) and up-regulating the expression of *DET2* (upstream gene) at DAA 50-70. The results show that exogenous JA significantly affects the expression of *ROT3* and *DET2* genes compared to EBR and other treatments because exogenous JA treatment increased their expressions in practically all sampling times aside from DAA 30, 70, and 80. In regard to the most important key genes related to BR biosynthesis (*BR6OX1* and *BR6OX2*), exogenous EBR up-regulated their expression levels at DAA 30, DAA 40, and DAA 60, as well, exogenous JA effectively promoted the expression of *BR6OX1* and suppressed the expression of *BR6OX2* at DAA 30 and DAA 40. Interestingly, the BR development in grape berries was dramatically reduced at DAA 80 by exogenous EBR or JA with the addition of Brz and DIECA, and compared with the control group, the expression of key BR biosynthetic genes (*BR6OX1* and *BR6OX2*) and BR metabolic gene (*BAS1*) were both down-regulated.

### JA biosynthesis and metabolism

**Figure 6:**
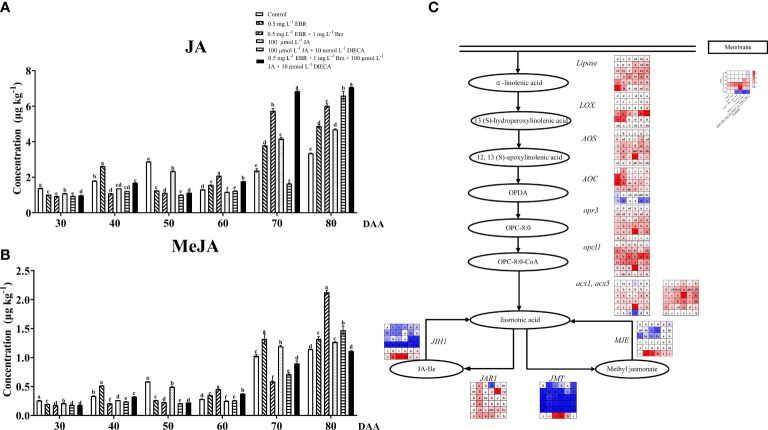
Effect of exogenous EBR, JA and their signaling inhibitors on JA/MeJA biosynthesis and metabolism. **(A, B)** Effect of exogenous EBR, JA and their signaling inhibitors on JA/MeJA contents in different treatment groups (Control, 0.5 mg L^-1^ EBR, 100 μmol L^-1^ JA, 0.5 mg L^-1^ EBR + 1 mg L^-1^ Brz, 100 μmol L^-1^ JA + 10 mmol L^-1^ DIECA, 0.5 mg L^-1^ EBR + 1 mg L^-1^ Brz + 100 μmol L^-1^ JA + 10 mmol L^-1^ DIECA) at six sampling stages (DAA 30, 40, 50, 60, 70, 80). **(A)** JA contents. **(B)** MeJA contents. The levels of JA and MeJA were expressed in the form of a histogram. Error bars = ± SE (n≥3). Significant differences among samples have been displayed by a–f letters (one-way ANOVA, P<0.05). **(C)** Expression of genes involved in JA/MeJA biosynthesis and metabolic pathways in different treatment groups (Control, 0.5 mg L^-1^ EBR, 100 μmol L^-1^ JA, 0.5 mg L^-1^ EBR + 1 mg L^-1^ Brz, 100 μmol L^-1^ JA + 10 mmol L^-1^ DIECA, 0.5 mg L^-1^ EBR + 1 mg L^-1^ Brz + 100 μmol L^-1^ JA + 10 mmol L^-1^ DIECA) at six sampling stages (DAA 30, 40, 50, 60, 70, 80). The expression levels of genes were remarked as a heatmap. Significant differences among samples have been displayed by a–f letters (one-way ANOVA, P<0.05).

The results showed that JA and MeJA in this investigation had a constantly increasing tendency with fruit ripening, as illustrated in **Figures 6A, B**. Exogenous EBR and JA treatments increased the contents of JA and MeJA at DAA 70 and DAA 80. Garrido-Bigotes et al. (2018) also described that the exogenous MeJA application increased the concentration of endogenous JA and MeJA in strawberries. Treatments containing Brz and DIECA decreased the JA and MeJA levels at DAA 30–50, respectively.

**Figure 6C** presents the expression levels of related genes in JA/MeJA biosynthesis and metabolism pathways. Regarding JA biosynthesis, we found that exogenous EBR and JA treatments regulated the expressions of *lipase*, *AOS*, *AOC*, *opr3*, *opcl1*, *acx1*, *acx5*, *JIH1* and *MJE* at DAA 60, as well as up-regulated the expression of *LOX* at DAA 30 and 80. Except for DAA 30, both exogenous applications (EBR and JA treatments) significantly increased the levels of *JAR1* (responsible for JA metabolism) and *JMT* (responsible for MeJA biosynthesis) throughout berry development. It’s worth emphasizing that the use of Brz enhanced JA metabolic genes (*JAR1* and *JMT*) expressions at DAA 80, and the use of DIECA enhanced their expressions at DAA 70 while decreasing the expression levels of JA biosynthesis-related genes (*LOX*, *opr3*, *acx1*, *acx5*) at DAA 80. Similar results were observed when MeJA was applied to peaches, which demonstrated that the JA biosynthesis gene was up-regulated, and PpWRKY45 played an important role in the up-regulation of JA biosynthesis-related genes (Ji et al., 2021). Additionally, MeJA up-regulates the expression of the JA biosynthesis genes (*BrLOX4*, *BrAOC3*, *and BrOPR3*), which contributed to the production of endogenous JA levels in Chinese cabbage (Tan et al., 2018).

Thus, we concluded that exogenous EBR and JA significantly increased the levels of JA and MeJA by inducing the expression of the majority of the genes involved in JA biosynthesis, metabolism, and MeJA biosynthesis.

### Endogenous phytohormones formation

In this study, the homeostasis of endogenous BR and JA in grape berries was certainly regulated by exogenous EBR and JA. At the same time, the changes in other phytohormones were still worth investigating. Therefore, another experiment was conducted to investigate the effects of exogenous EBR, JA, and their signaling inhibitors, on the content of other endogenous phytohormones in grape berries (**Figure S3**). Exogenous EBR increased IAA levels at DAA 50 (108.8%) and DAA 60 (129.1%) compared to the control group; however exogenous JA decreased IAA contents throughout the study (**Figure S3A**). Exogenous EBR and JA increased the ABA content at DAA 50 to 70 (**Figure S3B**). As for SA content, exogenous EBR and JA decreased their contents at DAA 30, 50 and 70 (**Figure S3C**). Moreover, exogenous EBR and JA increased GA_3_ contents at DAA 50 to 80 (**Figure S3D**). According to Heidari et al. (2021), exogenous BR encouraged the rise of endogenous ABA, IAA, and GA_3_ contents in tomatoes, while MeJA increased the rate of ABA and JA in both maize and citrus (Tayyab et al., 2020).

Cytokinins (CTKs) are another important class of phytohormones that regulates the differentiation and proliferation of plant cells (Xu et al., 2020). In this study, five kinds of CTKs were determined. Compared to the control group, exogenous EBR and JA increased ZT accumulation by 107.8% to 330.3% and 114.0% to 203.6%, respectively, at all sampling intervals other than DAA 50 (**Figure S3E**). As shown in **Figure S3F**, exogenous EBR and JA increased ZR contents only at DAA 70. EBR (DAA 40–70) and JA (DAA 70 and 80) treatment groups had higher concentrations of iP and iPA content than the control group **(** **Figures S3G, H** **)**. Furthermore, under both exogenous EBR and JA treatments, KT contents were higher than the control group at DAA 30 and 70 (**Figure S3I**). Tang et al. (2022) also reported that the application of exogenous MeJA significantly increased the total CTKs content in ‘Wuyunjing 24’ and ‘Ningjing’ 3 rice. In addition, we discovered both BRz and DIECA had inhibiting effects on the accumulations of stress resistance-related phytohormones (ABA, SA) and planted growth-related phytohormones (GA_3_, ZT, ZR, iP, KT) at almost all sampling stages.

It is a pity that the effects of exogenous BR and JA on the changes of endogenous phytohormones during grape berry development have not been systematically discussed. According to our results, most of the phytohormones (IAA, ABA, SA, GA_3_, ZT) accumulated during the late ripening stage, and CTKs-like phytohormones (ZR, iP, iPA, KT) peaked during the early ripening stage. Overall, exogenous EBR and JA promoted the accumulation of ABA, GA_3_, and some CTKs, while inhibiting the accumulation of IAA and SA during the late fruit ripening stages.

### Correlation between different phytohormones and berry quality

The interaction of endogenous plant hormones is essential for improving fruit quality (Fuentes et al., 2019; Kou et al., 2021; Rachappanavar et al., 2022). Unfortunately, the potential relationship between endogenous phytohormones (particularly BR) and the improvement of grape berry phenotypes has not been thoroughly studied. As a result, we conducted a correlation analysis between berry quality parameters and 12 different phytohormones identified in this study (**Figure S4**). In particular, we found that variations in physical parameters (such as single berry weight, longitudinal fruit diameter, and transverse fruit diameter) were strongly correlated with ABA, IAA, and ZT concentration; variations in biochemical characteristics were highly linked with BR, JA, MeJA, ABA, IAA, GA_3_, SA, ZT, and ZR contents; variations in antioxidants and fatty acid contents were highly positively correlated with JA, MeJA, ABA, IAA, GA_3_, SA, ZT and ZR activity, while slightly negative changes were correlated with the changes in iP, iPA and KT contents. Moreover, we observed that all phytohormones detected were negatively correlated with TA. Interestingly, GA_3_ and ZR indicated a strong positive correlation with almost all quality-related parameters (biochemical properties, antioxidant activity, fatty acids content). We found that the accumulation of GA_3_ during the late fruit ripening stage and the accumulation of ZR during the early fruit ripening period contributed to the biosynthesis of metabolites, the development of antioxidant capacity, and the enrichment of fatty acids in grape berries. Although different phytohormones contributed differently to the improvement of berry quality (GA_3_ and ZR had the biggest impact on the improvement of berry quality), they continued to be essential to the overall improvement of grape berry quality. Further research is required to determine how these phytohormone interactions and their molecular mechanisms affect the regulation of the enhancement of grape berry quality.

## Conclusion

In conclusion, exogenous EBR and JA application changed the endogenous phytohormones (especially raised the endogenous BR and JA enrichment), improved berries quality, increased antioxidant activity, sugars accumulation, and flavor improvement in the grapevine fruit known as ‘Ruidu Hongyu’. BR and JA signaling inhibitors could effectively reduce the promoting effects caused by exogenous EBR and JA. In the meanwhile, there was a close relationship between ABA/IAA/ZT and fruit expansion, BR/JA/MeJA/GA_3_/ZR and the improvement of biochemical characteristics, JA/MeJA/ABA/GA_3_/SA/ZR and the enhancement of antioxidant capacity, and JA/MeJA/IAA/GA_3_/ZT/ZR and the accumulation of fatty acids. Although different endogenous phytohormones contributed differently to berry appearance changes and the generation of various metabolites, they all continued to regulate the improvement of grape berry quality. As a result, spraying 100 μmol L^-1^ JA and 0.5 mg L^-1^ EBR before the veraison stage was an effective strategy to promote the post-harvest grape berry quality. Further research is needed to determine the molecular mechanisms by which BR and JA interact to regulate the production of endogenous phytohormones to increase fruit quality.

## Data availability statement

The original contributions presented in the study are publicly available. This data can be found here: https://doi.org/10.6084/m9.figshare.21119356.v1.

## Author contributions

JL implemented all experiments, data analyses, original draft writing, and figures drawing. HJ reviewed and revised the whole manuscript. ZW and LW ground samples and reviewed the manuscript. JH and YZ provided all experimental materials, and CM, SJ, and CZ reviewed and edited the manuscript. SW designed the experiments, provided ideas, and supervised this study. All authors contributed to the article and approved the submitted version.

## Funding

This study was supported by the project of developing agriculture by science and technology in Shanghai (2019-02-08-00-08-F01118), Science and Technology Project in North Jiangsu (SZLYG202007), Work station for characteristic grape industries in Wenping Xu plateau, Yunnan (Kunming) (YSZJGZZ-2021061), as well as the China Agriculture Research System of MOF and MARA.

## Acknowledgments

The authors sincerely appreciated the reviewers and editors for their careful revising and selfless assistance. Meanwhile, we were also grateful to those who selflessly provide help with this research.

## Conflict of interest

The authors declare that the research was conducted in the absence of any commercial or financial relationships that could be construed as a potential conflict of interest.

## Publisher’s note

All claims expressed in this article are solely those of the authors and do not necessarily represent those of their affiliated organizations, or those of the publisher, the editors and the reviewers. Any product that may be evaluated in this article, or claim that may be made by its manufacturer, is not guaranteed or endorsed by the publisher.

## Supplementary material

Supplementary Figure 1 The design idea and graphical abstract of the whole experiment. This figure covered all treatments involved, studies carried out as well as the results obtained.

Supplementary Figure 2 Effect of exogenous EBR, JA and their signaling inhibitors on grape berry antioxidant capacity. (A-D) Effect of exogenous EBR, JA and their signaling inhibitors on grape berry antioxidant activities in different treatment groups (Control, 0.5 mg L^-1^ EBR, 100 μmol L^-1^ JA, 0.5 mg L^-1^ EBR + 1 mg L^-1^ Brz, 100 μmol L^-1^ JA + 10 mmol L^-1^ DIECA, 0.5 mg L^-1^ EBR + 1 mg L^-1^ Brz + 100 μmol L^-1^ JA + 10 mmol L^-1^ DIECA) at six sampling stages (DAA 30, 40, 50, 60, 70, 80). (A) CAT activity. (B) POD activity. (C) SOD activity. (D) PPO activity. The activities of each antioxidant were expressed in the form of a histogram. Error bars = ± SE (n≥3). Significant differences among samples have been displayed by a–f letters (one-way ANOVA, P<0.05). (E, F) Effect of exogenous EBR, JA and their signaling inhibitors on membrane lipid peroxidation parameters in different treatment groups (Control, 0.5 mg L^-1^ EBR, 100 μmol L^-1^ JA, 0.5 mg L^-1^ EBR + 1 mg L^-1^ Brz, 100 μmol L^-1^ JA + 10 mmol L^-1^ DIECA, 0.5 mg L^-1^ EBR + 1 mg L^-1^ Brz + 100 μmol L^-1^ JA + 10 mmol L^-1^ DIECA) at six sampling stages (DAA 30, 40, 50, 60, 70, 80). (E) MDA contents. (F) Pro contents. The levels of each metabolite were expressed in the form of a histogram. Error bars = ± SE (n≥3). Significant differences among samples have been displayed by a–f letters (one-way ANOVA, P<0.05).

Supplementary Figure 3 Effect of exogenous EBR, JA and their signaling inhibitors on endogenous phytohormones contents in different treatment groups (Control, 0.5 mg L^-1^ EBR, 100 μmol L^-1^ JA, 0.5 mg L^-1^ EBR + 1 mg L^-1^ Brz, 100 μmol L^-1^ JA + 10 mmol L^-1^ DIECA, 0.5 mg L^-1^ EBR + 1 mg L^-1^ Brz + 100 μmol L^-1^ JA + 10 mmol L^-1^ DIECA) at six sampling stages (DAA 30, 40, 50, 60, 70, 80). (A) IAA contents. (B) ABA contents. (C) SA contents. (D) GA_3_ contents. (E) ZT contents. (F) ZR contents. (G) iP contents. (H) iPA contents. (I) KT contents. The levels of each phytohormone were expressed in the form of a histogram. Error bars = ± SE (n≥3). Significant differences among samples have been displayed by a–f letters (one-way ANOVA, P<0.05).

Supplementary Figure 4 The correlation analysis among different endogenous phytohormones and berry quality-related parameters. Total of 12 phytohormones were represented in the vertical row, 3 kinds of appearance parameters, 11 kinds of biochemical properties, 6 kinds of antioxidant-related substances and 10 kinds of fatty acids were represented in the horizontal row. The strength of the relational degree was indicated by bubbles size, in which a positive correlation was represented by red color and a negative correlation was represented by blue color.

Supplementary Table 1 Detection and quantification limits of phytohormones by HPLC.
