# Supplementary material for: Improving berry quality and antioxidant ability in ‘Ruidu Hongyu’ grapevine through preharvest exogenous 2,4-epibrassinolide, jasmonic acid and their signaling inhibitors by regulating endogenous phytohormones
Source: Front Plant Sci. 2022 Dec 2;13:1035022. doi: 10.3389/fpls.2022.1035022 (PMC9755660; doi:10.3389/fpls.2022.1035022)
Supplement: Supplementary Table 1 — Detection and quantification limits of phytohormones by HPLC. [file DataSheet_2.zip › Table 1.DOCX]

| **Phytohormones** | **Linear range** | **Calibration slopes** | **Correlation coefficient**  **(R^2^)** | **LOD**  **(μg L^-1^)** | **LOQ**  **(μg L^-1^)** |
| --- | --- | --- | --- | --- | --- |
| ABA | 0-100 | 208545 | 0.9993 | 1 | 3.33 |
| IAA | 0-100 | 14476 | 0.9992 | 0.71 | 2.34 |
| GA_3_ | 0-2000 | 396.21 | 0.9990 | 0.96 | 3.17 |
| SA | 0-1000 | 4703.3 | 0.9994 | 2.075 | 6.84 |
| JA | 0-100 | 8238 | 0.9998 | 0.38 | 1.25 |
| MeJA | 0-100 | 23025 | 0.9998 | 0.55 | 1.81 |
| KT | 0-100 | 470279 | 0.9992 | 0.59 | 1.94 |
| ZT | 0-20 | 106740 | 0.9998 | 0.8 | 2.64 |
| ZR | 0-20 | 63529 | 0.9999 | 0.73 | 2.41 |
| iP | 0-40 | 36591 | 0.9995 | 1.04 | 3.43 |
| iPA | 0-600 | 144636 | 0.9992 | 0.28 | 0.92 |

**Table S1** Detection and quantification limits of phytohormones by HPLC.
